# A Review of Exposure Assessment Methods in Epidemiological Studies on Incinerators

**DOI:** 10.1155/2013/129470

**Published:** 2013-06-12

**Authors:** Michele Cordioli, Andrea Ranzi, Giulio A. De Leo, Paolo Lauriola

**Affiliations:** ^1^Department of Bio-Sciences, University of Parma, Parco Area delle Scienze 11/a, 43124 Parma, Italy; ^2^Regional Reference Centre Environment & Health, Regional Agency for Environmental Protection in Emilia-Romagna, Via Begarelli 13, 41121 Modena, Italy; ^3^Hopkins Marine Station and Woods Institute for the Environment, Stanford University, 120 Oceanview Boulevard, Pacific Grove, CA 93950, USA

## Abstract

Incineration is a common technology for waste disposal, and there is public concern for the health impact deriving from incinerators. Poor exposure assessment has been claimed as one of the main causes of inconsistency in the epidemiological literature. We reviewed 41 studies on incinerators published between 1984 and January 2013 and classified them on the basis of exposure assessment approach. Moreover, we performed a simulation study to explore how the different exposure metrics may influence the exposure levels used in epidemiological studies. 19 studies used linear distance as a measure of exposure to incinerators, 11 studies atmospheric dispersion models, and the remaining 11 studies a qualitative variable such as presence/absence of the source. All reviewed studies utilized residence as a proxy for population exposure, although residence location was evaluated with different precision (e.g., municipality, census block, or exact address). Only one study reconstructed temporal variability in exposure. Our simulation study showed a notable degree of exposure misclassification caused by the use of distance compared to dispersion modelling. We suggest that future studies (i) make full use of pollution dispersion models; (ii) localize population on a fine-scale; and (iii) explicitly account for the presence of potential environmental and socioeconomic confounding.

## 1. Introduction

Incineration is one of the most common technologies for waste disposal [1]. The number of incineration plants in Europe has been constantly rising in the last years, in the effort to manage and treat an ever-increasing waste production according to the EU directives and minimizing landfill disposal [2]. As waste incineration releases in the atmosphere chemicals that are potentially toxic [3], there is increasing public concern about the possible adverse effects on human health caused by this waste management technology [4, 5].

The literature on health effects of waste incinerators is extensive and can be essentially classified into two groups: observational studies (i.e., epidemiological analyses) and simulation studies (i.e., health risk assessment). The first group includes studies that make use of a variety of statistical techniques to describe the potential relationship between the *observed* health status of the population and the exposure level from incinerators. The second group includes studies aimed at estimating the *expected* impact, in terms of health risk and/or number of sanitary cases, of a measured or simulated exposure to environmental contaminants [6–8]. 

Available epidemiological studies have been well reviewed in many published papers [9–11] and reports published by international agencies [12, 13]. However, the lack of a common framework for study designs makes the results of the different investigations on the health impacts hardly comparable and thus inconclusive. Poor exposure assessment is claimed as one of the main reasons of inconsistency of results in published studies [3, 9, 10, 13]. 

Exposure is generally defined as the contact between a stressor and a receptor and can be characterized either by direct (e.g., personal monitoring and biological markers) or indirect methods (e.g., environmental monitoring, modelling, and questionnaires) [14]. Although direct measures of exposure can be considered the best measures for assessing the effect of a specific substance on the target population, indirect measures of exposure (e.g., simulations of atmospheric dispersion) have greater utility for source emission assessment and control, since they are capable of linking population health to specific pollution emission sources [14]. These indirect methods have rapidly evolved in the last years [15], especially due to the increasing diffusion of the use of Geographical Information Systems (GIS) [16] and computer models to simulate atmospheric dispersion [17]. 

The aims of the present work were twofold: first, we wanted to investigate what methods and approaches are commonly used in the published literature to characterize exposure levels from waste incinerators; second, we wanted to assess, through a computer simulation study, how the classification of the expected exposure level may change as a function of the method used to estimate it.

The analysis was performed by using the literature database gathered within a project supported by the Emilia Romagna Region (North Italy) between 2007 and 2012 (MONITER Project) [18], to standardize environmental monitoring and health surveillance methods in areas characterized by the presence of incinerators and to evaluate the health status of populations around the eight incinerators of the region. 

Although the focus of the present work was on waste incinerators, the results of our analysis can be extended to any point source of atmospheric pollution [19] or more generally to contaminated sites, where the presence of multiple sources has to be taken into proper account.

## 2. Material and Methods

### 2.1. Literature Review

We analyzed papers referenced in previously published reviews on incinerator health effects [9–13, 20] and, additionally, searched for further references on MEDLINE, PubMed, Scopus, and Google Scholar by using a number of keywords combinations (e.g., “epidemiology,” “incinerator,” “adverse effect,” etc.). We focused our analysis only on observational epidemiological studies. Human biomonitoring [21, 22] and risk assessment studies [7, 8, 23] were not considered here. We excluded also studies on incinerator's workers [9] as the exposure pathway and levels can be completely different from those experienced by the population living around the incinerator plants. 

**Figure 1:**
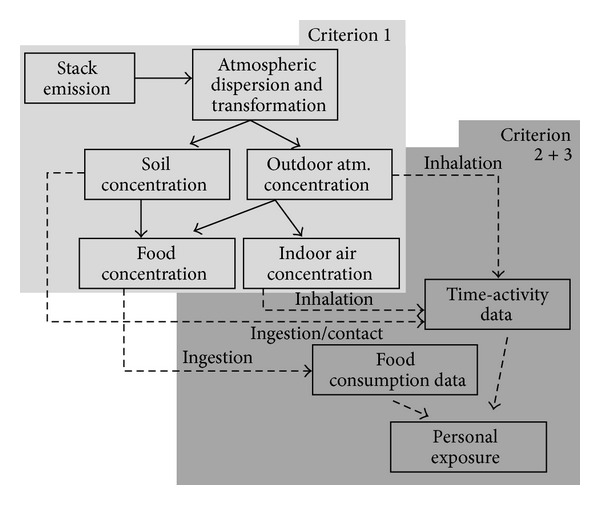
Conceptual model representing the principal impact pathways that determine exposure to atmospheric emissions from an incinerator. Contamination of drinking water is not represented.

The studies reviewed, rather than defining a relationship between environmental pollution and human health, aimed at evaluating the possible association with a specific industrial source of pollution (i.e., incinerators). The conceptual model for the emission-exposure pathways is sketched in Figure 1. Waste incineration epidemiological studies usually focus on gas stack emissions from the combustion process, while other possible sources of pollution (water discharges, ashes, smell emission, traffic, etc.) are not generally investigated [3]. After the emission from the incineration stack, pollutants dispersion in the atmosphere depends upon a number of physical and environmental variables such as stack height, wind speed and direction, temperature, and atmospheric stability. Some gases may undergo various chemical transformations, and part of the contaminants may eventually settle down on a variety of surfaces such as soil, vegetation, and water. Concentrations in the atmosphere and in soil may be either directly inhaled, ingested, or absorbed through dermal contacts or they can enter the agricultural food chain [24]. The actual exposure to potentially hazardous contaminants is thus determined by the time spent by various sectors of the population in different environments (outdoor, indoor at home, or at work) and could be due to inhalation, ingestion of contaminated water or food, and dermal contact with contaminated vectors (e.g., soil, water) [25]. Since incinerators are potential sources of persistent pollutants (e.g., dioxins, heavy metals, etc.) [3], ingestion can represent a relevant exposure pathway. 

Exposure to pollutants has conceptually at least three dimensions, namely, (i) the intensity of exposure, which depends among the other things upon the concentration level of contaminants in different media; (ii) space, as both population density and concentration of contaminants are spatially heterogeneous; (iii) time, which is the duration and variability of exposure, as this determines the total amount of contaminant that has been eventually ingested, inhaled, or absorbed through dermal contacts [14]. Exposure assessment reconstructs the relationship between receptors and locations and between locations and the presence and amount of a certain risk factor. Accordingly, we reviewed the selected literature focusing only on the approaches used to define the exposure level and classifying them on the basis of three criteria (Table 1):

1. the approach used to define the intensity of exposure to the emission source (3 categories);
2. the scale at which the spatial distribution of the exposed population was accounted for (3 categories);
3. whether temporal variability in exposure was considered or not in the published study (2 categories).

The combination of all categories can result in a total of 18 possible methods of exposure assessment and was hereafter referred to as “*x*.*y*.*z*,” where *x* represents the method used to estimate expected intensity, *y* the method used to estimate population distribution, and *z* whether the exposure was variable or not in time. For example, a published study classified as “2.3.1” means that the exposure level was evaluated as a function of the distance from the source, population distribution in the territory was assessed by using exact residential address location, and exposure was fixed in time.

Exposure assessment methods were categorized only on the basis of the exposure variables actually used in the epidemiological model. As discussed afterward, some studies reported additional information (such as measured concentrations of pollutants in various media) useful to interpret or support exposure model outcomes, even though this information was not used in statistical calculations. 

Another important element of the exposure assessment process is the control of confounding factors, that is, variables that may hide or enhance the measure of effect [26, 27]. These factors can be socioeconomic (e.g., people living in industrial areas near incinerators may be more deprived) or environmental (e.g., frequently incinerators are located in areas with high pollution from other industrial sources and traffic). 

For each reviewed study we analysed also whether and how confounding factors were accounted for. Since evaluation of confounding factors can follow a variety of approaches, we decided not to include this aspect as a fourth criterion in our classification scheme. Nevertheless we thoroughly comment on the role of confounding factors as well as their importance in epidemiological studies in the discussion.

### 2.2. Case Study: Parma

To understand how the choice of one or another approach of Table 1 may ultimately affect the estimated exposure, we run a simulation case study based on real data from an epidemiological surveillance program for a new incinerator that is under construction in the city of Parma (Italy). 

The data used to simulate the effect of alternative methods of exposure assessment were as follows:

- location of the stack of the incinerator;
- exact location of the address of residence for 31,019 people living around the incinerator (circle of 4 km of radius);
- boundaries of the 2001 Italian census blocks for the area, as defined by the Italian National Institute of Statistics;
- the results of an atmospheric dispersion model for PM_10_ emitted from the incinerator.

Geographic coordinates of addresses were provided by the local registry office. Atmospheric dispersion was simulated using the ADMS Urban model [28], a second generation quasi-Gaussian model already employed in other studies on health effects of incinerators [29–31]. Since the study area is located in a flat plane, this model was judged suitable to compute long-term average concentration and deposition [32]. 

We used PM_10_ as a tracer for the complex mix of pollutants emitted by the incinerator, after a test on various types of pollutant. The aim of the simulation was to determine a geographic gradient of exposure inside the study area: this spatial gradient is mainly determined by the incinerator's characteristics and atmospheric conditions, while it is only poorly dependent on the pollutant's properties. 

We used five years of hourly meteorological data (2005–2010) from the nearest meteorological station (about 4 km from the plant) and source characteristics from the authorized project (i.e., stack height: 70 m; gas temperature: 150°C; PM_10_ emission flux: 231 mg s^−1^) to calculate average hourly concentrations at ground level (ng m^−3^) and average hourly deposition (ng m^−2^ h^−1^) of PM_10_ over the period 2005–2010 on a regular 200 m receptor grid. Calculated concentrations were interpolated (using quadratic inverse distance weighting) to obtain continuous maps (Figure 3). 

For each individual, we evaluated residential time-invariant exposure to the incinerator using the following methods:

1. distance between census block centroid and incinerator (CBDI, method 2.2.1);
2. distance between exact address location and incinerator (ADDI, method 2.3.1);
3. average concentration and deposition inside the census block of each address (CBCO and CBDE, method 3.2.1);
4. concentration and deposition at the address location (ADCO and ADDE, method 3.3.1).

We then contrasted the results of using alternative approaches for the assessment of the exposure level for each individual in the sample. Exposure variables were categorized in 5 classes (i.e., 1: lowest exposure, 5: highest exposure) using quintiles of each variable distribution. Thus, each exposure class contain approximately the same number of subjects. Only for address distance from the incinerator we defined also a second categorization using regular buffers, as done in the majority of published studies [33–35].

Concentration and deposition estimates based on dispersion models are affected by their own degree of uncertainty and should be possibly ground trued with field measurements and/or experiments. A previous validation study conducted in France [32] demonstrated that this kind of models provide a reliable proxy for incinerator exposure in simple terrain such as the area under study: we here assumed that simulated concentrations represent the closest estimate to the actual exposure.

Therefore we evaluated the degree of exposure misclassification using two-way tables and Cohen's kappa test of agreement [36, 37]. Cohen's kappa was calculated using quadratic weighting to assign less importance to misclassification between adjacent classes and higher importance to other misclassifications. 

## 3. Results

### 3.1. Literature Review

A total of 41 studies published between 1984 and January 2013 were identified by the literature search. Table S1 in Supplementary Material available online at http://dx.doi.org/10.1155/2013/129470 (Supplementary Information) reports the resulting categorization of exposure methods and other relevant information for each study. The column “covariates” lists the confounding factors that were evaluated in each study.

**Figure 2:**
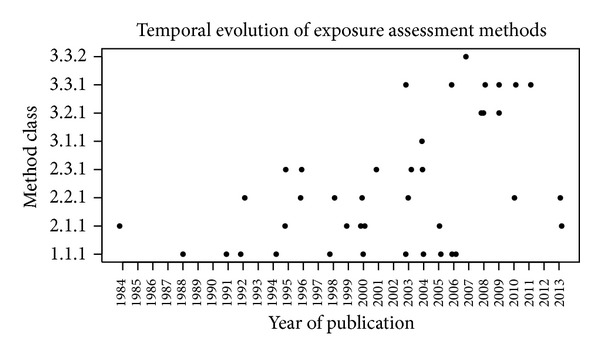
Temporal evolution of exposure assessment methods. Methods are classified according to Table 1 and sorted in the *y*-axis from the less precise to the best one.

Figure 2 represents the evolution of methodologies in time, based on the year of publication. Methods on the *y*-axis are sorted from the less precise to the best one. 

With reference to the first classification criterion, that is, method used to assess exposure intensity, 19 studies (46%) used a measure of distance, both on a continuous scale and more commonly by defining concentric areas with arbitrary radius. In some cases [38–41] also wind direction was used to introduce some spatial anisotropy in exposure. Lee and Shy [42] used distance to define exposed communities but developed also a longitudinal study using daily PM_10_ measurement from fixed air monitors. One study [43] analysed spatial clustering of disease cases: since the analysis was based on the position of the community of residence, we classified this method as 2.1.1. One study [41] presented multiple assessment methods: presence/absence of the incinerator, distance from the plant, and an exposure index based on distance, wind direction, and time spent outdoor by people.

11 studies (27%) used atmospheric dispersion models to define population exposure. Generally models were used to estimate long-term average atmospheric concentrations at ground level, although one study used cumulated depositions [44]. Two studies [29, 45] used also heavy metals as indicator of exposure, while all the others used dioxins. 

The remaining 11 studies (28%) used a qualitative definition of exposure to contrast the health status of communities/municipalities with and without incinerators. One study [46] developed quantitative indicators to classify municipalities, using emission inventories for dioxin from incinerators.

All the published studies used the residence as the place where exposure to atmospheric pollution occurs (criterion no.2). Nevertheless, different levels of detail were used in defining residence location. The majority of the papers (*n* = 19, 46%) considered the municipality or community of residence (e.g., postcode sector, school, hospital, etc.), 12 studies (29%) used the exact geographic coordinates of the address of residence, and 10 (24%) used the full postcode or census unit. 

Finally, all the published literature, with one exception [47], defined exposure proxies that did not account for temporal variability in population spatial distribution and incinerators' emissions (criterion 3) that is, they considered the residence at the time of diagnosis, at enrolment, or the longest residence of the subject. Residential histories and changes in exposure intensity (e.g., as a consequence of changes in combustion and gas depuration technologies) were not accounted for in the other examined studies. 

Overall, Figure 2 shows a trend of improvement in the quality of exposure assessment during the examined years, although three studies published after 2010 still used linear distance as the exposure proxy.

### 3.2. Results of the Simulation Case Study in Parma

**Figure 3:**
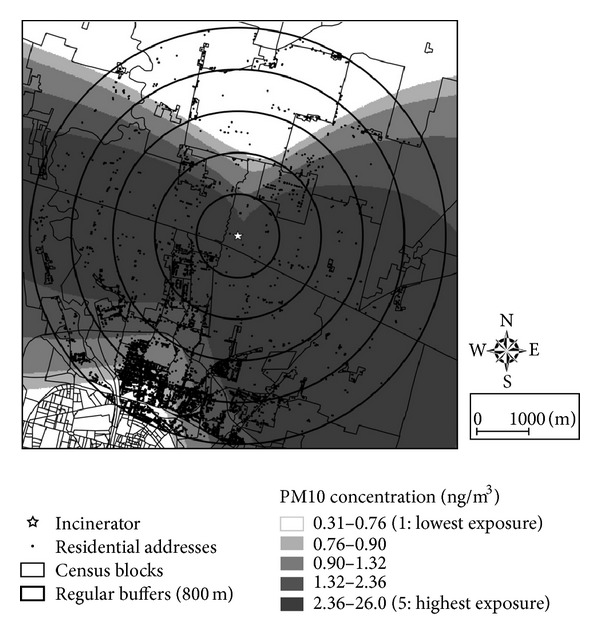
Representation of the area considered in the case study of Parma.

**Figure 4:**
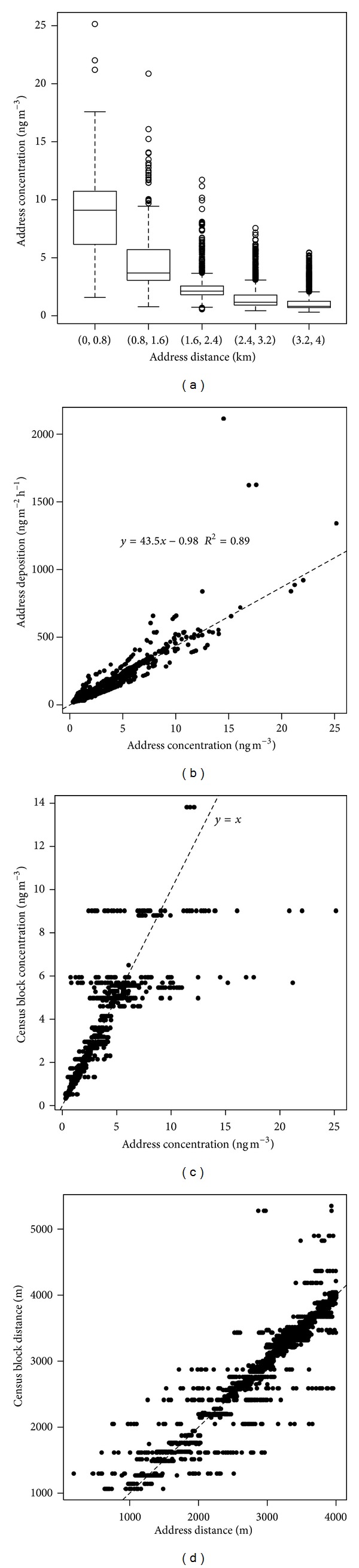
Results of exposure assessment by using different methodologies. (a) Variability of residential address concentration (ADCO) inside each regular 800 m buffer. Boxes represent the interquartile range (IQR), the horizontal line inside the box is the median value, and the whiskers extend to 1.5 times the IQR from the box. (b) Relationship between ground concentration (ADCO) and deposition (ADDE) at addresses location. The line represents the linear regression model. (c) Relationship between simulated concentrations evaluated at exact address (ADCO) and at census block level (CBCO). The line represents the 1 : 1 relationship. (d) Relationship between distance of the exact address (ADDI_1_) and distance of the census block centroid (CBDI). The line represents the 1 : 1 relationship.

Figure 3 reports the map of the census blocks around the incinerator under construction in Parma, its location (the star), the location of the sample of resident people used in the present study (small black dots), the expected PM_10_ concentration as simulated with the ADMS model, and the regular, 800 m wide, circular buffers around the emission source.  Figure 4 contrasts the results of alternative approaches to assess exposure level in terms of intensity (simulated concentration versus distance from the emission sources) and accuracy in residence location. 

Table 2 shows Cohen's kappa indices of agreement between concentration maps and other exposure assessment methods. The table reports also the share of individuals over the 31,019 samples assigned to the same class of exposure, the share of individuals classified in an adjacent exposure class, and that of individuals classified into two or more classes apart. High kappa values are encountered when concentrations and depositions are considered, while comparison between concentration and distance approaches gave worst results when distance is categorized on regular concentric circles.

## 4. Discussion

### 4.1. Evaluation of Exposure Intensity (Criterion 1)

The majority of the papers reviewed in the present study appear to suffer from poor exposure characterization. A relevant part of these papers (28%) used qualitative definitions of exposure (e.g., presence/absence of the source or anecdotic presence of pollution). These methods cannot account for the complexity of impact pathways described in Figure 1 nor for the heterogeneity in the exposure level that is normally expected as a consequence of the uneven distribution of the resident population and of the anisotropic dispersal of pollutants in the atmosphere. For instance, in the simulation case study we ran in Parma, the use of method 1.1.1 (presence of the incinerator in the municipality) would not allow us to discriminate between different levels of exposure and, therefore, all the 30,019 people in our sample (as well as the remaining 158,660 inhabitants of Parma) would be all classified as highly exposed, which would probably not be the case.

Epidemiological analyses carried out on a significant number of municipalities still represent a valuable instrument for public health tracking since they can evidence disease clusters in some regions that must be studied further. Even though any departure of disease incidence in large communities from background levels has to be taken very seriously, it is very difficult to use this type of evidence to infer about the role of specific emission sources (i.e., an incinerator), as many other potential confounding factors might exert a significant effect, particularly in highly urbanized areas. Moreover, the risk of false positive and, to a greater extent, false negative results, common to all exposure assessment methods, can be exacerbated when epidemiological data are averaged out on a vast territory with large internal differences in the exposure levels, as in method 1.1.1.

Almost half of the studies used distance to measure exposure. This is certainly a substantial improvement with respect to just an absence/presence evaluation, as contamination from an atmospheric emission source (e.g., air, soil, and locally produced food) is generally expected to decrease with distance. However, the assumption of isotropy in atmospheric dispersion of contaminants could lead to remarkable errors in exposure assessment. Many features of the emission source (e.g., stack height, gas flow temperature and velocity, and pollutant concentration) and of the local environment (e.g., local meteorology, topography, and land use) determine where and how far stack emissions disperse and how ultimately enter different environmental compartments. 

In our simulation study carried out for the Parma incinerator, the distance method assigns the same exposure level to people resident in the northern and eastern parts of the territory around the emission stack, even though simulations showed that concentrations are expected to be higher along the east-west direction than to the north-south one (Figure 3).

Because of the anisotropic dispersion of pollutants in the atmosphere, the expected PM_10_ concentrations at the residence address vary wildly inside each 800 m wide buffer around the incinerator (Figure 4(a)). Consequently, the use of distance from the emission source as a proxy of actual concentrations could cause a high degree of misclassification (Table 2).

The use of well-tuned atmospheric dispersion models allows a substantial improvement in the estimation of exposure level, especially if carried out along with a fine scale estimation of the spatial distribution of the vulnerable population. Anyway, atmospheric pollution models are themselves affected by a considerable level of uncertainty [48] depending upon assumptions on actual atmospheric conditions, reconstruction of wind fields, and type of dispersion processes, including the possibility of simulating chemical transformation which are known to be highly relevant for the formation of tropospheric ozone and secondary fine particulate matter. 

A significant number of the published papers analysed in the present study provided only a limited information on the atmospheric model: generally there was no discussion about the type of model used, the type and source of meteorological data, model adequacy to represent complex morphological natural or urban landscape and/or wind calms, and the assumptions made about pollutant's emission rates and physical-chemical properties.

Only few studies explicitly acknowledged limitations in the modelling approach used. For example, instead of adopting a different dispersion model as suggested by the same authors in a previous study [32], in Viel et al.'s [49] a part of the study area was excluded because dispersion model results were judged unreliable in that area. Another study [50] used maps of ground level concentrations estimated on the basis of emissions and meteorological data, but no dispersion model was cited. Almost all the studies used dioxins as an impact indicator: dioxins represent a family of 210 congeners, each one with different physical-chemical characteristics: no study clearly explained how these chemicals were treated in the model (e.g., using 2,3,7,8-TCDD congener properties). Moreover, some studies did not report a clear definition even of the most basic variables used to measure exposure, that is; averaging time for concentrations [31, 45, 51] or distinction between concentrations and depositions to ground [31]. As shown in our case study ground level atmospheric PM_10_ concentrations and depositions from a point source have very similar patterns with some significant departure, nevertheless the choice of one or the other measure of exposure should be at least discussed, related to the main route of exposure considered. All these pieces of information are important to judge the quality of the exposure assessment process, its uncertainties, and to allow comparability and reproducibility of methods.

Regardless of how detailed, accurate and advanced the model to simulate atmospheric dispersion is, it is still only a part of the impact pathways described in Figure 1. All the studies implicitly assumed that inhalation represents the principal exposure pathway, while no published literature measured or modelled the possible exposure through ingestion of contaminated food or contact with contaminated soil.

No study used measured levels of pollution in different media (e.g., atmosphere, soil, and food) as the exposure variable in the epidemiological model, except for one work [42] that used also measured 24 h average PM_10_ concentrations in each community as a predictor for pulmonary function, although there were no differences in average levels between communities defined *a priori* as exposed and not exposed. Many studies presented information on measured levels of pollution [43, 52–54], but these data were not included in the statistical model. This is not surprising, as it is very difficult to discriminate the contribution of single-point sources to the observed concentrations levels. The latter, in fact, invariably depend the contribution of several other confounding emission sources [55, 56], especially if they are located in urbanized areas with intense traffic or industrial activities. Thus, indirect measures of exposure obtained through modelling represent a valid alternative useful to identify the possible role of a specific emission source.

### 4.2. Evaluation of Receptor's Exposure (Criteria 2 and 3)

The actual exposure of an individual to the pollutants emitted by an incinerator may occur in different environments and last a variable amount of time. All published studies used the residence as the place where exposure to atmospheric pollution occurs (criterion 2). Notably, one study [57] considered also the location of workplace of studied subjects. 

Residence location can be determined with various degrees of precision. The majority of revised studies (48%) used community level to determine residence location (i.e., town, municipality, postcode sector, and school). In this way the same exposure level is assigned to large groups of population, but this assumption was rarely discussed and no measures of exposure variability inside groups were reported. Thus, it was impossible to evaluate the degree of ecological bias [58] that is, how well the variation in risk between groups with different average exposure applies to the variation in risk between individuals.

Some studies used census block or full postcode for determining residence position. The dimension of these blocks may vary greatly depending on the location: normally these blocks are smaller in populated areas but may become very large in other rural zones. Moreover, no information was generally given about blocks extension, and it was difficult to compare very different blocks types like Small Area Health Statistic Unit (SAHSU) [35], UK census postcode system [59], or UK Lower Layer Super Output Areas (LSOA) [60]. In our case study census blocks had an average area of 0.4 km^2^ (min: 968.4 m^2^; max: 6.3 km^2^) and contained on average 26 addresses (min: 1; max: 130): both address distances and concentrations vary widely inside some census blocks (Figures 4(c) and 4(d)). This was true especially for more exposed areas, since the incinerator is located in a less densely populated area with large census blocks. This aspect could lead to different degree of errors in exposure assignment, that increase with the level of pollutant or proximity to the incinerator.

The most precise way to locate residences is to address geocoding: this procedure assigns a couple of geographic coordinates to each address. Errors in address positioning depend on the quality of the database used but is generally in the order of tens to hundreds meters [61, 62], thus small in comparison with the use of census blocks or full postcode. 

In future studies maximum disaggregation of data, to maximise information and minimize potentially differential ecological biases [63], is thus recommended.

The use of residence as exposure location is a very common assumption in environmental epidemiology since it is easily derived and there is evidence that people normally spend a great part of their time inside their residences, for example, on average 69% [64] and 80% [65]. Nevertheless, home location may not well represent total exposure because people may experience shorter but more intense exposures outside home, and residence is a proxy only for inhalation exposure and does not account for indirect pathways [66] (Figure 1). Although this technique has well-known limitations, it is often the only method available, particularly for large populations or for reconstructing historical exposures.

Temporal variability in exposure is an issue rarely explored in the reviewed studies. Temporal variability may result both from changes in source emissions over time or from residential mobility of the population and may be a cause of incorrect exposure assignment [67, 68]. Only one published study [47] explicitly accounts for historical exposure variability by reconstructing residential histories and evolution of dioxin emissions from the sources considered. However the exposure indicator chosen (i.e., the average exposure over time) may introduce some bias: since emissions from the sources considered were progressively reduced starting from the 1990s, the average exposure value decreases with the increase of exposure duration. A better indicator could have been cumulative exposure, that is, the sum of the annual exposure concentration over the exposure duration. One study [29] considered the modification of incinerator emissions over time indirectly, without considering changes in the final statistical model, but evaluating how the morphology of fallout maps was similar in time.

Although difficult to achieve because of data unavailability, especially for studies on old incinerators, in future studies efforts should be developed in reconstructing residential histories and variability in incinerator's emission over time, at least as a sensitivity analysis for the model.

### 4.3. Exposure Misclassification and Confounding Factors

Almost all papers used categorical definitions of exposure (i.e., exposure classes). One issue rarely discussed is the rationale behind the choice of cut-off values used to classify continuous variables. In the absence of toxicological reference values for this type of exposure, in our case study we used a criterion expected to make the results of the statistical analysis more stable and reliable, that is, having roughly the same number of exposed individuals in each class. In reviewed studies *a priori* cutoffs of exposure were generally chosen without an explicit justification [33–35, 51]. 

When categorical exposure variables are measured with error, they are said to be misclassified. Misclassification can be differential or nondifferential with respect to disease status of an individual person [26], the latter being more probable in reviewed studies and generally leading to risk estimations biased toward the null. Nevertheless, in presence of more than two exposure categories, non-differential misclassification can move estimates of risk away from null and disrupt exposure-response trends [69]. 

Our case study showed that

- for exposure measures based on distance a relevant part of the population may be classified in the wrong exposure category (assuming that dispersion model better represents real exposure), with relevant percentages of subjects moving by more than one category;
- the use of census blocks to identify the residence may introduce a certain degree of differential misclassification since the error is higher in more exposed areas and lower for less exposed.

Both these factors may bias risk estimates away from the null or modify exposure-response trends. 

Sometimes, the degree of error in exposure assessment can be evaluated with a validation study [70], that is, comparing modelled exposure with “gold-standard” measurement of exposure collected for a random subsample of the population, such as direct measurement of individual exposure. In practice, since no such gold standard is generally available, we recommend researchers to conduct sensitivity analyses on exposure assessment [71] and discuss the magnitude of error that may be present in their data.

Another issue that is only partially dealt with in reviewed literature is confounding. Confounding occurs when a risk factor different from the exposure variable under study causes bias in the estimation of association between exposure and disease, due to its differential distribution in exposed and non exposed groups [72]. Various confounding factors may affect a study on incinerators' health effects, that is, socioeconomic differences (e.g., poverty, occupation), personal lifestyles (e.g., alcohol, smoke), and presence of other sources of pollution. 

Many reviewed studies did not account for any confounder in the epidemiological model [33, 47, 59, 73–77]. Some studies collected information about personal lifestyles or socio-economic status directly through questionnaires [38–40, 51, 78, 79]. Unfortunately the use of questionnaires and surveys is unfeasible for large populations; thus a large part of the studies did not consider personal lifestyles but included socio-economic indicators (e.g., deprivation indexes) evaluated at municipality/census block of residence [29, 30, 35, 44, 45, 49, 80, 81]. These indexes are generally constructed based on census statistics.

Of particular concern is the general lack of information about environmental confounding. Many of the pathologies under study have been associated with various atmospheric pollutants (e.g., PM_10_, NO_*x*_, etc.) or specific anthropogenic sources (e.g., road traffic, industrial emissions). Often, waste incinerators are located inside industrial areas or near other major sources of pollution. In our case study, for example, the incinerator is located inside the industrial area of Parma, at about 200 m from a national highway that crosses the study area east-west (i.e., the prevalent wind directions). As a result, most exposed subjects, as identified by the dispersion model, were also more exposed to other sources of pollution. It will be difficult to correctly identify the possible health effect of this incinerator, unless we have some information about the difference in population exposure to other sources between the exposed and nonexposed groups. Only few studies included information about environmental confounders. Biggeri et al. [79] used measured particulate depositions from the nearest monitoring station, Cordier et al. [45] used proxies for the presence of industrial activities and road traffic at community level, and two studies [31, 44] used proxies for traffic and industrial pollution at census block level. Notably, one recent study [29] used atmospheric dispersion models to estimate pollution concentrations at the address of residence from other local sources of atmospheric pollution (road traffic, industrial plants, and heating). This represents a notable improvement since the confounding factor was evaluated with the same spatial resolution as exposure to the incinerator. 

As the quantitative contribution of well-managed modern incinerators to total pollution levels in a study area and to baseline health risks is expected to be low, we suggest to draw a careful attention to other local sources of pollution and to implement multisite studies on large populations where feasible.

## 5. Conclusions

We reviewed 41 articles from the literature with the main aim of retrieving information for the definition of an exposure assessment protocol to be used in a large study on health effects of pollution due to incinerators (MONITER project). 

Overall, our analysis showed a trend of improvement in exposure assessment quality over time, with a massive use of dispersion models in exposure assessment after year 2003.

Nevertheless, the lack of a common framework for exposure assessment is demonstrated by the use of a variety of methods, also in recent papers, with different quality of epidemiological findings and difficulties in comparisons of results.

In most of the selected studies the characterization of exposure can be significantly improved by using more detailed data for population residency and better simulation models. Recent development of informative systems and high availability of environmental and demographic data suggest the use of dispersion models of pollutants emitted from a source, combined with precise methods of geographic localizations of people under study, as the state of the art method to assess exposure of population in epidemiological studies. Considerations about residential mobility, temporal variations in pollution emissions, latency period of investigated diseases, and treatment of environmental and sociodemographic confounders can improve exposure assessment accuracy.

All these aspects of exposure assessment are particularly relevant as most of environmental conflicts usually arise from the evaluation of the contribution of the various pollution sources to the overall contamination. 

## Supplementary Material

Table S1: lists all the reviewed studies and reports the resulting classification of exposure assessment methods and the list of confounding factors considered, together with other relevant information useful to evaluate the quality of the study.

## Figures and Tables

**Table 1:** Classification of exposure assessment methods.

Category	Description
Criterion 1: definition of exposure intensity	

1	Qualitative (e.g., presence/absence of the source/contamination in an area)
2	Distance from the source (e.g., linear distance)
3	Dispersion models (e.g., average annual atmospheric concentration)

Criterion 2: definition of population distribution	

1	Municipality/community/postcode sector
2	Census unit/full postcode
3	Exact residential address location

Criterion 3: temporal variability	

1	Time-invariable (i.e., fixed) exposure
2	Time-variable exposure (e.g., residential history and/or variability in emissions from the source)

**Table 2:** Evaluation of the agreement between concentration maps and other exposure assessment methods. Quadratic weighted Cohen's kappa and percentages of subjects classified in the same exposure class or in different classes.

Comparison exposure	Weighted kappa^a^	Matching subjects	Misclassification in adjacent categories	Misclassification in >1 class apart
ADCO versus ADDE	0.91	69.6%	29.3%	1.1%
ADCO versus CBCO	0.97	89.2%	10.5%	0.3%
ADCO versus CBDE	0.90	70.0%	27.8%	2.2%
ADCO versus ADDI_1_	0.61	38.9%	45.1%	16.0%
ADCO versus CBDI	0.60	40.2%	44.5%	15.3%
ADCO versus ADDI_2_	0.35	25.4%	39.8%	34.8%

ADCO: address concentration (quintiles), ADDE: address deposition (quintiles), CBCO: census block concentration (quintiles), CBDE: census block deposition (quintiles), ADDI_1_: address distance (quintiles), ADDI_2_: address distance (regular 800 m buffers), CBDI: distance between census block centroid and incinerator. ^a^all kappa with *P* < 0.001.
