# Supplementary material for: A Review of Exposure Assessment Methods in Epidemiological Studies on Incinerators
Source: J Environ Public Health. 2013 Jun 12;2013:129470. doi: 10.1155/2013/129470 (PMC3694556; doi:10.1155/2013/129470)
Supplement: Supplementary file 1 — Table S1: lists all the reviewed studies and reports the resulting classification of exposure assessment methods and the list of confounding factors considered, together with other relevant information useful to evaluate the quality of the study. [file 129470.f1.pdf]

SUPPLEMENTARY INFORMATION for:

A review of exposure assessment methods in epidemiological studies on incinerators

Michele CORDIOLI [1,2] , Andrea RANZI [2] , Giulio A. DE LEO [3] , Paolo LAURIOLA [2]

[1] Department of Bio- Sciences, University of Parma, Parma, Italy

[2] Regional Reference Center Environment & Health, Regional Agency for Environmental Protection in Emilia-Romagna, Modena, Italy

[3] Hopkins Marine Station and Woods Institute for the Environment, Stanford University, California , US

Table S1 - Reviewed studies, classified by exposure method (see Table 1 in the paper). See paper bibliography for references.

**Study**	**Outcome**	**Country**	**Population**	**Industrial sources**	**Exposure measure in the model**	**Other exposure information**	**Covariates**	**Class**
Zimrou et al. (1984) [82]	Consumption of medicines for respiratory symptoms	France		1 industrial incinerator	Distance of the area of residence from the plant (200,1000,2000 m)			**2.1.1**
Lloyd et al. (1988) [83]	Twinning frequency	United Kingdom	258 mothers 470 twins	1 MSWI<br>1 CWI	Residence in postcode sectors at low VS medium/high risk (qualitatively based on wind direction and distance)	Polychlorinated hydrocarbons in soil	Maternal age	**1.1.1**

Study	Outcome	Country	Population	Industrial sources	Exposure measure in the model	Other exposure information	Covariates	Class
Hsiue et al. (1991) [78]	Pulmonary function in children	Taiwan	382 children	Open wire reclamation incineration	Children from schools in 3 impacted areas (qualitatively defined) VS 1 school in control area.	Measures of NO_2 and SO_2 , higher in impacted areas.	Questionnaire (passive smoking)	**1.1.1**
Williams et al. (1992) [59]	Sex ratio	United Kingdom	3,576 births	2 incinerators	Birth in a postcode district classified as at risk VS low risk (qualitatively, based on wind direction, topography, people perception and soil PAH concentrations)			**1.1.1**
Elliott et al. (1992) [80]	Larynx and lung cancer	United Kingdom		10 incinerators of solvents and olis	Distance of postcode zone of residence (SAHSU) from the sources, observed VS expected in circles of 0-3 and 3-10 km		Socioeconomic status	**2.2.1**
Gray et al. (1994) [54]	Childhood asthma or allergy	Australia	713 exposed, 626 control children	High temperature sludge burning	Two regions close to the incinerators VS one region without incinerators	air pollution measures of $NO_{2,}$, SO_2, HF e PM (no differences between areas)		**1.1.1**
Barbone et al. (1995) [38]	Lung cancer mortality	Italy	755 cases, 755 controls	1 incinerator, 1 iron foundry, shipyard, city centre	Distance of the last residence from the industrial sources + wind direction	PM deposition measurement in 28 stations + PM concentrations from 4 stations	Questionnaire (smoke, occupation)	**2.3.1**
Shy et al. (1995) [39]	Chronic and acute respiratory effect	North Carolina, US	6,963 participants	1 MSWI,<br>1 BWI<br>1 HWI	Residence in a community within 2x5 km ellipse (oriented along principal wind direction) from the incinerator VS upwind outside 5 km	12h pollution measurement in each community, CMB estimation of incinerator contribution	Questionnaire	**2.1.1**

Study	Outcome	Country	Population	Industrial sources	Exposure measure in the model	Other exposure information	Covariates	Class
Biggeri et al. (1996) [79]	Lung cancer	Italy	755 cases, 755 controls	1 incinerator, 1 iron foundry, shipyard, city centre	Distance and direction between the last residence and the sources	PM concentrations	Smoke, occupation, PM concentrations	**2.3.1**
Elliott et al. (1996) [35]	Cancer incidence	United Kingdom	14 million people	72 MSWI	Distance from postcode zone of residence (SAHSU), observed VS expected in 0-3km, 0-7.5 km,0.5-1-2-3-4.6-5.7-6.7-7.5 km radius buffers		Deprivation score (SAHSU)	**2.2.1**
Michelozzi et al. (1998) [34]	Cancer mortality	Italy	341,389 residents	1 landfill, 1 incinerator, 1 refinery	Distance between centroid of census block and centroid of the industrial area, SMR for 3-8-10km buffers + 1km buffers		Deprivation score (census block)	**2.2.1**
Rydhstroem et al. (1998) [73]	Twin deliveries	Sweden	17,067 twin deliveries	14 incinerators	Expected VS observed twinning in municipalities before/after the activation of incinerators			**1.1.1**
Lee et al. (1999) [42]	Pulmonary function	North Carolina, US	480 residents	1 MSWI 1 BWI 1 HWI	Residence in a community within 2miles from the incinerator VS outside 2 miles + Longitudinal analysis with PM_{10}	24h PM_{10} concentration in each community (no differences)	Questionnaire	**2.1.1**

Study	Outcome	Country	Population	Industrial sources	Exposure measure in the model	Other exposure information	Covariates	Class
Tusscher et al. (2000) [74]	Orofacial cleft in children	Nederland	8,803 children	1 chemical open burning site	Pregnancies at a clinic near the burning site VS pregnancies at a clinic 12km far	Mother's residences dispersion around the burning site (qualitative). Tons of waste burned. Some measures of environmental contamination		**1.1.1**
Mohan et al. (2000) [40]	Respiratory symptoms	North and South Carolina, US	4,025 subjects	1 MSWI<br>1 BWI<br>1 HWI<br>1 IWI	4 communities within 2x5 (or 8x5) km ellipse VS 4 communities 5 to 25 km upwind from exposed communities	PM_{10} average concentration per community	Questionnaire (demographic, house characteristic)	**2.1.1**
Knox (2000) [84]	Childhood cancer	United Kingdom	22,458 children	70 MSWI<br>307 BWI<br>460 hazard waste landfills	Distance (at birth VS at death) of the postcode of residence from sources			**2.2.1**
Viel et al. (2000) [43]	Soft tissue sarcoma Non Hodgkin Lymphoma	France	110 STS 803 NHL, 176 HL	1 incinerator	Cluster analysis based on residence in a "canton"	Information on dioxin emissions and concentrations in milk		**2.1.1**
Hu et al. (2001) [41]	Pulmonary function	North Carolina, US	10,187 subjects	1 MSWI<br>1 BWI<br>1 HWI	Residence in a comunity with incinerator VS with no incinerator (>5km) + distance from the address of residence to the incinerator + exposure index (function of distance, direction, n. days downwind, n. hours outside home)		Questionnaire (smoking, house characteristics)	**2.3.1**

Study	Outcome	Country	Population	Industrial sources	Exposure measure in the model	Other exposure information	Covariates	Class
Comba et al. (2003) [33]	Soft tissue sarcoma	Italy	37 STS cases 171 controls	1 IWI 1 oil refinery, 1 paper and 1 chemical industry	Distance of the main residence from the incinerator, buffer 1 km (<2, 3, 4, 5, >5km)			**2.3.1**
Dummer et al. (2003) [85]	Adverse pregnancy outcomes	United Kingdom	244,758 births	3 crematorium, 4 incinerators	Distance of mother residence to the plants		Social class, year, birth order, multiple births	**2.2.1**
Floret et al. (2003) [51]	Non Hodgkin Lymphoma	France	225 cases (match 10:1)	1 MSWI	PCDD/F average atmospheric concentrations from dispersion modelling (APC3 Gaussian), 4 classes of exposure (very low to high)	Dioxin concentrations in soil [32]	Education, occupation, social class and household-based indicators	**3.3.1**
Fukuda et al. (2003) [46]	Mortality from major diseases	Japan	803 municipalities		Residence in a municipality with an incinerator VS without. Indices of incineration activity at municipality level derived from emission inventory.		7 socio-economic indicators	**1.1.1**
Cordier et al. (2004) [45]	Congenital anomalies	France	2,872 communities with less than 50'000 inhabitants	70 incinerators	PCDD/F concentrations in most populate areas from dispersion model (POLAIR, Gaussian), multiplied by the num. of years of functioning of the plant. 3 classes of exposure		Pop. density and income at community level, local traffic, presence of industries, mother age	**3.1.1**

Study	Outcome	Country	Population	Industrial sources	Exposure measure in the model	Other exposure information	Covariates	Class
Parodi et al. (2004) [52]	Lung cancer mortality	Italy	118749 residents	1 waste incinerator, 1 power plant, 1 lead oxide industry	Residence in industrial area VS residence in urban/semi-urban areas	Measures of heavy metals in lichens higher in industrial areas	Age, deprivation index	**1.1.1**
Tango et al. (2004) [53]	Adverse reproductive outcomes	Japan	225,215 live births, 3,387 foetal deaths, 835 infant deaths	63 incinerators	Distance of maternal residence from incinerators, 1 km buffers	Measurement of dioxin in soil for two plants	Stratification by vital statistics (mother age, weight, father employment)	**2.3.1**
Miyake et al. (2005) [86]	Allergic disorders and general symptoms	Japan	450,807 children in 996 schools	37 MSWI	Distance from the school to the nearest incinerator		Socioeconomic status, healthcare access in the municipality	**2.1.1**
Biggeri et al. (2005) [75]	Soft tissue sarcoma, Non Hodgkin Lymphoma	Italy		1 MSWI	Residence in the municipality with the incinerator VS residence in other municipalities 80 km around		Age	**1.1.1**
Bianchi et al. (2006) [77]	Non Hodgkin Linfoma	Italy	1,830 NHL	25 incinerators	Residence in a municipality with an incinerator VS residence in municipalities 30-50 km around		Age	**1.1.1**
Biggeri et al. (2006) [76]	Non Hodgkin Lymphoma	Italy	1,119 NHL	17 incinerators	Residence in a municipality with an incinerator VS residence in municipalities 50-80 km around			**1.1.1**

Study	Outcome	Country	Population	Industrial sources	Exposure measure in the model	Other exposure information	Covariates	Class
Tessari et al. (2006) [50]	Neoplasias	Italy	188 sarcomas, 134 HL, 774 NHL, 24,184 cancers	Various industrial sources of PCDD/F (3 incinerators)	Concentration of PCDD/F at the residence address from a model for all sources in the area. 4 exposure categories.		Sex, age	**3.3.1**
Zambon et al. (2007) [47]	Sarcoma	Italy	171 cases 405 controls	26 incinerators 7 industrial plants	PCDD/F dispersion model (ISC3, Gaussian,), average concentration weighted by length of each residence. Reconstruction of 40 years of emission data.			**3.3.2**
Viel et al. (2008) [49]	Breast cancer	France	434 cases 2,170 controls	1 MSWI	PCDD/F dispersion model (APC3, Gaussian) concentration at census block. 4 classes of exposure		Socioeconomic at census level	**3.2.1**
Viel et al. (2008b) [31]	Non Hodgkin Lymphoma	France	3974 NHL cases	13 incinerators	PCDD/F dispersion model (ADMS3 Gaussian), cumulative deposition to soil, median of all receptors in each census block		Pop. density, urbanisation, socio-economic level, traffic and industrial pollution	**3.2.1**
Vinceti et al. (2008) [57]	Abortion and congenital anomalies	Italy	23 cases	1 MSWI	PCDD/F dispersion model (ISC3, Gaussian + Spray, Lagrangian), concentration at mother address and place of work, 2 classes of increasing exposure			**3.3.1**

Study	Outcome	Country	Population	Industrial sources	Exposure measure in the model	Other exposure information	Covariates	Class
Goria et al. (2009) [44]	Cancer	France	135,000 cases	16 incinerators	PCDD/F dispersion model (ADMS3, Gaussian) cumulative depositions to soil, median of receptors at census block level.		Pop. density, rural/urban indicator, socio-economic score, road traffic pollution and other polluting industries	**3.2.1**
Vinceti et al. (2009) [87]	Congenital anomalies	Italy	228 cases	1 MSWI	PCDD/F and heavy metal dispersion modelling (Windimula, Gaussian), concentration at mother address. 3 classes of exposure.		Mother education and age	**3.3.1**
Cordier et al. (2010) [30]	Urinary tract birth defect	France	324 cases<br>226 controls	21 MSWI	PCDD/F and heavy metal dispersion modelling (ADMS3), concentration and deposition at home address, 3 classes of exposure.	Consumption of local foods enhance risk in the most exposed areas while lower risk in less exposed areas	Deprivation index, pop.density, questionnaire, other pollution sources at municipality level, old incinerators	**3.3.1**
Federico et al. (2010) [81]	Cancer incidence	Italy	16,443 cancer cases	1 MSWI	Distance between residence census block centroid and incinerator's census block centroid (2-3.5-5 km buffer). Cluster analysis		Deprivation index at census block	**2.2.1**
Ranzi et al. (2011) [29]	Mortality and morbidity	Italy	31'374 individuals	1 MSW incinerator	Heavy metal dispersion model (ADMS Urban, Gaussian), concentrations at the address of residence, 4 classes of exposure plus analysis population inside 3.5 km buffer VS Regional population	Measurement of various pollutants in air and soil determined heavy metals as preferred indicator of MSWI impact	Dispersion model for pollution from other sources (industry, traffic, heating), deprivation index at census block level	**3.3.1**

Study	Outcome	Country	Population	Industrial sources	Exposure measure in the model	Other exposure information	Covariates	Class
Garcia-Pèrez et al. (2013) [88]	Cancer mortality	Spain	8098 municipalities	129 installations for waste treatment<br>14 incinerators	Distance from the town centre to the facility. Near (<5km) VS far analysis.		Socioeconomic status at municipality level	**2.1.1**
Reeve et al. (2013) [60]	Cancer and all cause mortality	United Kingdom		5 incinerators (> 150'000)	Distance between the incinerator and the centroid of census unit (LSOA) of residence. Comparison of exposed (<10km) and control (>10 km) areas.		Age, sex, deprivation index at census level	**2.2.1**

ABBREVIATIONS

MSWI = municipal solid waste incinerator; CWI = chemical waste incinerator; PAH = polycyclic aromatic hydrocarbons; NO_2, SO_2 = nitrous and sulphur dioxide; SAHSU = small area health statistic unit; HF = fluoridic acid; PM = particulate matter; BWI = biomedical waste incinerator; HWI = hazardous waste incinerator; CMB = chemical mass balance model; IWI = industrial waste incinerator; PCDD/F = dioxins and furans; LSOA = Lower Layer Super Output Area
